# Role of topical application of iced slush in the development of phrenic nerve palsy after cardiac surgery

**DOI:** 10.1186/1749-8090-10-S1-A8

**Published:** 2015-12-16

**Authors:** Ahmed Oliemy, Sanjeet Singh, John Butler

**Affiliations:** 1 Golden Jubilee National Hospital, Glasgow, UK

## Background/Introduction

Phrenic nerve palsy (PNP) is a well-recognized complication after cardiac surgery which might occur as a result of topical myocardial cooling, direct injury or ischemia. It can result in deleterious respiratory effects especially in children and in patient with chronic obstructive pulmonary disease which adversely affect recovery and increase the length of ICU and hospital stay. Topical application of iced slush has been used for decades in hypothermic cardiac surgery, the effect of the direct contact between the iced slush and phrenic nerve might increase the incidence of nerve palsy and diaphragmatic dysfunction. Recently normothermic cardiac surgery has been employed largely to avoid the adverse effects of hypothermia.

## Aims/Objectives

To prove the relation between topical myocardial cooling and phrenic nerve palsy.

## Method

This study is a retrospective observational study looking at the incidence of phrenic nerve palsy in consecutive 196 cardiac surgery patients, 102 of whom had iced slush applied topically with moderate hypothermic cardiopulmonary bypass (28-32) -ICE group- and 94 had nothing applied with a normothermic cardiopulmonary bypass (37) -WARM group. Phrenic nerve palsy was suspected in patients with raised hemidiaphragm on Chest radiographs. Subsequent CXRs were taken prior to discharge and at the 6 week post-operative clinic for resolution.

## Results

In the ICE group PNP was observed in 10.8% (11 of 102 patients) versus 0% (0 of 94 patients) in the WARM group (p = 0.0010), there were no significant difference between both groups regarding the mean age, preoperative respiratory risk factors, and aortic cross clamp time. Logistic Euroscore was 4.07% for the ICE group and 2.33% for the WARM group (p = 0.0016), total bypass time was 76.5 minutes for the ICE group versus 86 minutes for the WARM group (p = 0.016). Length of postoperative ventilation was significantly higher in the ICE group (7 hours) than the WARM group (5 Hours) (p = 0.054), length of ICU and HDU stay were similar in both groups.

## Discussion/Conclusion

The use of iced slush during heart surgery might increase the incidence of postoperative phrenic nerve palsy with an impact on the length of postoperative ventilation.

